# Suppressive effects of dental pulp stem cells and its conditioned medium on development and migration of colorectal cancer cells through MAPKinase pathways

**DOI:** 10.22038/ijbms.2021.58273.12946

**Published:** 2021-09

**Authors:** Elham Nikkhah, Fatemeh Kalalinia, Mitra Asgharian Rezaee, Zahra Tayarani-Najaran

**Affiliations:** 1 Medical Toxicology Research Center, Mashhad University of Medical Sciences, Mashhad, Iran; 2 Biotechnology Research Center, Pharmaceutical Technology Institute, Mashhad University of Medical Sciences, Mashhad, Iran; 3 Department of Toxicology and Pharmacology, Faculty of Pharmacy, Kerman University of Medical Sciences, Kerman, Iran; 4 Pharmaceutical Research Center, Institute of Neuropharmacology, Kerman University of Medical Sciences, Kerman, Iran; 5 Targeted Drug Delivery Research Center, Pharmaceutical Technology Institute, Mashhad University of Medical Sciences, Mashhad, Iran

**Keywords:** Apoptosis, Colonic neoplasms, Conditioned medium, Dental pulp, MAP kinase cascade, Stem cells

## Abstract

**Objective(s):**

Mesenchymal stem cells (MSCs) extensively interact with cancer cells and other stromal cells in the tumor microenvironment. However, the role of MSCs in colorectal cancer (CRC) development and metastasis is controversial. Strong evidence demonstrated that conditioned medium (CM) obtained from MSCs regulates main cellular functions such as proliferation, differentiation, migration, and communication due to its cell secretomes. This study was designed to determine the inhibitory effect of dental pulp stem cells (DPSC) and its extracted conditioned medium (DPSC-CM) in CRC progression.

**Materials and Methods:**

The inhibitory effects of DPSC-CM on growth, apoptosis, and migration of CRC cells were evaluated by resazurin, flow cytometry of propidium iodide (PI) stained cells, and wound closure assay, respectively. Western blotting detected the expression of MAPKinase and apoptotic proteins. Also, the homing ability of DPSCs and the invasion ability of CRC cells under indirect co-culture were assayed by the Boyden chamber assay.

**Results:**

DPSC-CM reduced the viability and induced the apoptosis of CRC cells significantly. Western blot analysis confirmed the increase in cytochrome C, phospho-JNK/SAPK to JNK/SAPK ratio, cleaved-caspase 8 and 3 in treated CRC cells with DPSC-CM, and decrease in phospho-ERK (P44/42 MAPK) to ERK (P44/42 MAPK) ratio, which are involved in induction of apoptosis and growth inhibition of cancer cells with minimal change in normal cells. Also, DPSCs could migrate (homing ability) to Caco_2_ and SW48 cells significantly.

**Conclusion:**

To sum up, DPSC-CM had significant apoptotic and growth inhibitory effects on the CRC cells through the MAPKinase and apoptosis signaling pathways.

## Introduction

One of the most common cancers in the world is colorectal cancer (CRC), which is the third cause of death due to cancer worldwide (1). CRC development is a multi-step process wherein genetic alteration leads to subverted intestinal epithelium cell division and differentiation. This alteration occurs through gene mutations and epigenetic alterations that lead to inactivation of tumor suppressors and activation of oncogenes (2, 3). 

Mesenchymal stem cells (MSC) are multipotent mesenchymal stromal cells, which can home to the sites of inflammation, injury, and tumor (4). These kinds of stem cells can infiltrate into the tumor tissues and interact with a variety of cells in the tumor microenvironment (5). MSCs are easily transfected and relatively non-immunogenic (6). Different mechanisms have been suggested for inhibiting tumor growth by MSCs including anti-angiogenic activities, expression of IFN-β, and inhibition of the Wnt/β-catenin signaling pathway. Inhibition of ERK and activation of JNK as a part of MAPK cascade may have a role in differentiation and apoptosis of CRC cells, which control the developmental intestinal neoplasm (7).

In addition, other mechanisms such as inhibition of extracellular signal-regulated kinase (ERK) and activation of c-jun N-terminal kinase/ stress-activated protein kinases (JNK/SPAK) have been proposed (8-10). Therefore, MSCs seem to be suitable candidates for cell-based therapies for cancers (5). 

While some evidence shows tumor progression by MSCs, others show MSCs could suppress tumor growth (8, 10-13). Using the MSC-conditioned medium (MSC-CM) would be a suggested solution to prevent this contradiction. MSC-CM is the medium in which the stem cells are cultured and contain the secreted factors such as secretome, microvesicles, and exosomes. Additionally, a variety of extracellular matrix proteins, active cytokines, tissue remodeling enzymes, chemokines, growth factors, hormones, extracellular matrix proteins, and matrix remodeling enzymes have also been reported in MSC-CM using proteomic techniques (14, 15). It has been shown that MSC-CM could inhibit immune responses and some cancer progression such as human breast and lung cancer cells. Therefore, MSC-CM can be applied as an effective and practical acellular therapy and a new procedure for cancer inhibition (10, 16-18). 

Dental pulp stem cells (DPSCs), which are obtained by noninvasive procedures from dental pulp tissue, have similar therapeutic potential to other kinds of MSCs. They have been used to treat some diseases such as type 1 diabetes, neurological diseases, immunodeficiencies, and bone and cartilage disorders. Therefore, dental pulp could be a valuable noninvasive alternative source of MSCs for regenerative therapies (19, 20). In the present study, we aimed to evaluate the inhibitory effects of DPSCs and their conditioned medium (DPSC-CM) on colorectal adenocarcinoma as a cost-effective and non-invasive procedure to treat or inhibit the progression of CRCs.

## Materials and Methods

***Materials***

Resazurin sodium salt (Sigma), fluorescent probe propidium iodide (PI), and Calcein-AM fluorescence dye were obtained from Sigma (Germany); Collagenase type 1, Dispase, alpha modified Eagle’s medium (α-MEM), fetal bovine serum (FBS), penicillin/streptomycin (100 units/ml penicillin and 100 µg/ml streptomycin) and trypsin 0.25% / EDTA 0.1% were from Gibco (UK); Dimethyl sulfoxide (DMSO) and Crystal violet were from Merck (Germany); all antibodies were from Cell Signaling Technology (USA); antibodies were rabbit anti-cytochrome C polyclonal Ab (#4272), rabbit anti-Pro and Cleaved caspase 3 monoclonal Ab (#9565), rabbit anti-Pro and Cleaved caspase 8 monoclonal Ab (#4790), rabbit SAPK/JNK (#9252) and Phospho SAPK/JNK (#9251) polyclonal Ab, rabbit anti-ERK polyclonal (#9102) and rabbit anti-ERK Phospho ERK monoclonal Ab (#94370), rabbit anti-β-catenin monoclonal Ab (#8480), mouse anti-β-actin monoclonal Ab (#3700) and rabbit anti-IgG HRP-linked Ab (#7074) and mouse anti- IgG HRP-linked Ab (#7076). 

***Cell lines***

Two human CRC cell lines with different invasive capacities (Caco_2_ and SW48) and Human umbilical vein endothelial cells (HUVEC) were purchased from Pasteur Institute of Iran (IPI). Caco_2_ is a chromosomal instability (CIN) phenotype of human CRC. The prevalence of this phenotype, which is associated with a poor prognosis, is 80–85% of CRC. SW48 is a microsatellite instability pathway (MIN) CRC cell line with a 15–20% prevalence of CRC. SW48 cells are associated with proximal tumor location, lower staging and high-grade differentiation (21, 22). All cell lines were cultured in α-MEM media containing FBS 10% (v/v) and penicillin/streptomycin 1% and incubated at 37 °C, 5% CO_2_, and 95% humidity. 

***Isolation and detection of dental pulp stem cells (DPSCs)***

All procedures of this study were approved by the Ethics Committee of Mashhad University of Medical Sciences (No. 1396.154). Human DPSCs were isolated from third molars taken from healthy donors (n=6) who were 18–28 years old in the Clinic of Dentistry, School of Dentistry, Mashhad University of Medical Sciences, Mashhad, Iran. Informed consent was taken from all donors. Teeth were placed into the sterile container containing cold PBS buffer and were transferred to the laboratory at 4 °C. Dental pulp was removed from the pulp chamber and cut into 1–2 mm pieces under sterile conditions. Then, the dental pulp was incubated with collagenase (3 mg/ml) plus dispase (4 mg/ml) solutions for 30–40 min at 37 °C and centrifuged at 600 g for 5 min. The cell pellet was cultured in 12.5 cm^2^ culture flasks containing α-MEM medium supplemented with FBS (10%) and penicillin/streptomycin (1%) and incubated at 37 °C in a humidified atmosphere with 5% CO_2_. The medium was changed at 70% confluence after 10 days to remove non-adherent cells and every 2 or 3 days. The cells were passaged at approximately 70% confluence with trypsin/ EDTA and sub-cultured at a ratio of 1:2 (23). DPSCs were characterized for MSC-associated CD markers using flow cytometry. DPSCs were positive for the expression of CD29, CD44, CD90, and CD105, and negative for hematologic markers, CD34 and CD45 (24, 25). Moreover, DPSCs have been differentiated to the osteoblast and adipocyte (data not shown) (26, 27). 

***Production of DPSC-conditioned medium (DPSC-CM)***

DPSCs (passage 3-5) were cultured in 75 cm^2^ culture flasks with indicated cell count: 5000 (5K), 10000 (10K), 20000 (20K), and 40000 (40K) cells/cm^2^ (Table 1). After one day, the cells were washed 3 times with PBS and incubated with 8 ml FBS free α-MEM at 37 °C and 5% CO_2_. The conditioned medium was collected and centrifuged at 1200 × g for 5 min (to eliminate cell debris) after 2, 3, and 4 days (henceforth referred to as 2 d, 3 d, and 4 d, respectively). Finally, the conditioned mediums were frozen until use (16, 17, 28).

***Analysis of the effect of DPSC-CM on the cell viability***

Caco_2_, SW48 and HUVEC (5 × 10^3^) cells were seeded in 96-well culture plates. After 24 hr, the medium was removed, cells were washed 3 times with PBS and cultured with DPSC-CM or FBS free medium as an untreated control. The positive control group was treated with 5-FU (250 µM), which is applied clinically to treat the CRC. The cell viability was assessed after 2, 3, and 4 days by adding resazurin reagent and incubating at 37 °C for 4 hr. The absorbance was measured at 600 nm via a Synergy H4 Multi-Mode microplate reader (BioTek, Winooski, USA). Wells without cells served as the blank control and each group had three replications. 

***Apoptosis assay of CRC cells treated with DPSC-CM***

Caco_2_, SW48, and HUVEC cells (10^5 ^cells) were seeded in 24-well plates and incubated with CM-40k-4d for 3 days at 37 °C and 5% CO_2_. FBS free medium and 5-FU (250 µM) was used as negative and positive control, respectively. Then, the cells were trypsinized, suspended in 400 μl of a hypotonic PI buffer (50 μg/ml PI in 0.1% sodium citrate plus 0.1% Triton X-100) and incubated at room temperature in the dark for 30 min. After that the cells were analyzed by a FACS Scan flow cytometer (BD Biosciences, CA, USA). Sub G1 peak in the flow cytometry histogram of PI stained cells indicated the apoptotic cells (29).

***Wound migration assay***

The effect of DPSC-CM on the migration of the CRC cells was assessed by the wound migration assay. CRC cells (10^5^) were plated in a 24-well plate at 37 °C and 5% CO_2 _to reach 90% confluence. The monolayers of cells were scratched with a 100 µl micropipette tip. The cells were washed twice with PBS to remove the debris and incubated with CM-40K-3d and CM-40K-4d. FBS-free medium was used as control. Wounds were imaged using inverted microscopy at 10 X magnification at 0 hr and three days after incubating at 37 °C and 5% CO_2_. The wound area was analyzed with Image J software, version 1.50 I, and all data normalized according to the control (30-32).

***Western blotting analyses***

Expression levels of cytochrome C (Cyt C), Pro and Cleaved caspase 3, pro and cleaved caspase 8, JNK/SAPK and phospho JNK/SAPK, ERK and phospho ERK, JAK and phospho JAK, STAT and phospho STAT, β-catenin and PARP were determined by immunoblot analysis in Caco_2_, SW48, and HUVEC cells treated with DPSC-CM. Cells (2×10^6^) were cultured in 75 cm^2^ culture flasks and incubated with (CM-40K-4d) for 72 hr at 37 °C, 5% CO_2_. FBS free α-MEM medium was considered as control. Afterward, the cells were lysed by lysis buffer and total protein in the samples was quantitated by the Bradford reagent. Supernatant proteins (20 μl) were separated by 10 or 12% SDS–PAGE gel (related to the molar weight of considered protein) and transferred onto a polyvinylidene fluoride (PVDF) membrane. Membranes were blocked with 5% bovine serum albumin (BSA) for 2 hr and incubated with primary rabbit antihuman antibodies (1:1000 dilution) overnight at 4 °C in the dark or mouse anti-β-actin Ab (1:1000 dilution) for 2 hr. Then membranes were incubated with anti-rabbit or anti-mouse IgG HRP-linked Ab as secondary Ab (1:3000 dilution) for 1 hr in the dark. The bands were detected using Geldoc Alliance, version 4.7. Band densities were analyzed using Gelquantnet V.4.0 Gel Analysis Software and were normalized by the density of β-actin bands (11, 31, 33).

***Indirect co-culture system***

Indirect co-culture systems were used to observe intercellular interactions between cells with no direct physical contact which clarifies the reaction between the cell types. Different cell types were separated by culture chambers, filters, or gels in the indirect co-culture systems. Transwell or Boyden chamber co-culture plate is one of the standard methods for filter separation of cells. The properties of cells, which are cultured in the upper and lower chamber of Transwell were changed through exosome activity (34-36).

***Apoptosis assay of CRC cells indirectly co-cultured with DPSCs***

To assay the indirect effect of DPSCs on the apoptosis of CRC cells, DPSCs (1.2×10^4 ^cells/ml) were cultured in the upper chamber and Caco_2_, SW48, and HUVEC (1.2×10^5 ^cells/ml) were plated in the lower chamber of a 24-Transwell plate with 0.4 µm pore size, which prevents cell migration through the pores. DPSCs were co-cultured with Caco_2_, SW48, and HUVEC and incubated at 37 °C, 5% CO_2_ for 3 days. Thereafter, the cells in the lower chamber were harvested by trypsin (0.25%)/ EDTA (0.1%) and suspended in 400 μl of PI hypotonic buffer and analyzed by a FACS Scan flow cytometer (BD Biosciences, CA, USA). Sub G1 peak in the flow cytometry histogram of PI stained cells indicated the amount of apoptosis (16).

***DPSCs homing assay***

To assess the effect of CRC cells on homing of DPSCs, DPSCs (4×10^4^ cells/ml) were seeded on the upper chamber of a 24-Transwell plate with 8 µm pore size. This specific pore size of the membrane permits cells to pass through the pores, which is needed for migration (34). Caco_2_, SW48, and HUVEC cells (2×10^5^ cells/ml) were seeded in the lower chamber of the 24-Transwell plate. The plates were incubated at 37 °C and 5% CO_2_ for 3 days (12). Control wells contained FBS-free α-MEM in the lower chamber. Then, the inserts were removed and their upper surface was cleaned by a swab to remove non migrated cells. DPSCs that migrated to the underside of the inserts were fixed with methanol and stained with 0.1% crystal violet solution for 10 min. The crystal violet was dissolved in glacial acetic acid and its absorbance was recorded at 570 nm using a Synergy H4 Multi-Mode microplate reader (BioTek, Winooski, USA) (11). 

***CRC cells invasion assay***

To assess the effect of DPSCs on invasion of CRC cells, DPSCs (2×10^5 ^cells/ml) were cultured in the lower chamber of a 24-Transwell plate. Caco_2_ and SW48 (2×10^4^ cells /ml) were plated on the insert of the 24-Transwell plate with 8 µm pore size and placed on the lower compartment. The control contained FBS free α-MEM medium in the lower chamber. Transwell plates were incubated at 37 °C and 5% CO_2 _for 3 days. Cancer cells that migrated to the underside of the filter were fixed with methanol and stained with crystal violet. The optical density of crystal violet at 570 nm was recorded by Synergy H4 Multi-Mode microplate reader after dissolving (BioTek, Winooski, USA)(12).

***Statistical analyses***

All of the experiments were carried out in triplicate and the results were analyzed by prism 8. The significance of differences was evaluated by one-way and two-way ANOVA assay followed by Tukey’s test. All data were shown as mean ± SD and significance was expressed as *P<0.05, **P<0.01, and ***P<0.001.

## Results

***The effect of DPSC-CM on the cell viability***

**Figure 1:**
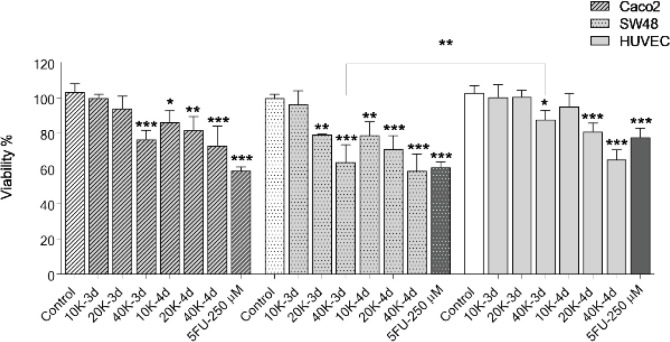
Effects of DPSC-CM on cell viability. Caco_2_, SW48, and HUVEC cells were treated with DPSC-CM for 3 and 4 days and viability was assayed by resazurin. Data are expressed as mean ± SD of three independent experiments in triplicate (*P<0.05, **P<0.01, ***P<0.001) compared with untreated control. 5-FU was used as a positive control

DPSC-CM: Dental pulp stem cell-conditioned medium; HUVECs: Human umbilical vein endothelial cells

To evaluate the inhibitory effect of DPSC-CM on proliferation of CRC cells, the cell viability was quantified by resazurin assay (Figure 1). Treatment with DPSC-CM collected from a higher density of stem cells could effectively inhibit the growth of CRC cells after 3 days. DPSC-CM decreased the viability of cancer cells beginning at 20K in SW48 (79±0.4%) and 40K in Caco_2_ (76±5.2%) after 3 days and increased to 58.5±2.2% and 73±10.9% for Caco_2_ and SW48, respectively with CM-40K-4d after 4 days with minimal effect on HUVEC as a normal cell. Moreover, the difference in the viability of SW48 treated with CM-40K-3d in comparison with HUVEC was significant (*P*=0.04).

***Effect of DPSC-CM on the apoptosis of CRC cells***

**Figure 2:**
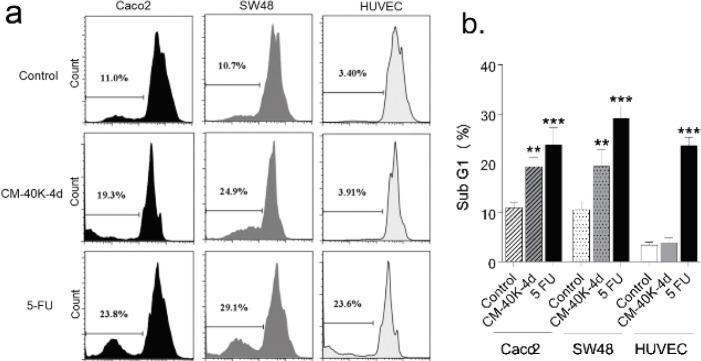
Effect of DPSC-CM on the apoptosis of CRC cells. A. Flow cytometry histogram of PI stained CRC and normal cells treated with DPSC-CM B. Data are expressed as mean ± SD of three independent experiments, ***P<0.001 and **P<0.01 compared with untreated control

DPSC-CM: Dental pulp stem cell-conditioned medium; CRC: Colorectal cancer

To detect the sub G1 peak of the apoptotic cells, CRC and normal cells were treated with CM-40K-4d for 3 days and analyzed by PI apoptosis assay. Sub G1 peak in the flow cytometry histogram of PI stained cells indicated the percentage of apoptotic cells. As indicated in Figure 2, CM-40K-4d induced apoptosis significantly in both CRC cells compared with control with minimal change in normal cells. 

***Effect of DPSC-CM on the CRC cells migration ability***

**Figure 3:**
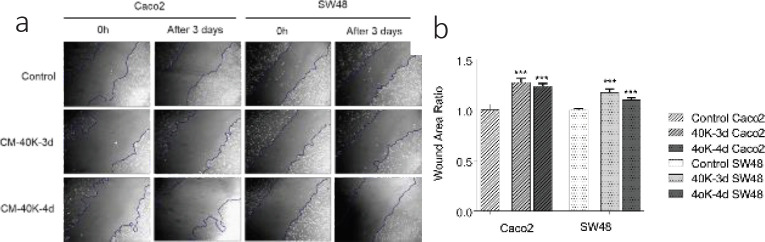
Effect of DPSC-CM on the migration of CRC cells evaluated by the wound migration assay. A. Caco_2 _and SW48 representative images of wound migration assay immediately after scratching and after 72 hr in the presence of FBS free medium (control), CM-40K-3d, and CM-40K-4d. Original magnification 10 X. B. Wound area in the presence of CM-40K-3d and CM-40K-4d for 3 days compared with the 0 hr. Data are expressed as mean ± SD of three independent experiments in triplicate samples. ***P<0.001 compared with the 0 hr

DPSC-CM: Dental pulp stem cell-conditioned medium; CRC: Colorectal cancer

The effect of CM-40K collected after 3 and 4 days on migration, was assessed after 3 days using light inverse microscopy and the area of wound closure was quantified by Image J software. Results showed that the wound area changes were significantly increased in CRC cells treated with CM-40k-3d and CM-40k-4d compared with the untreated control after 3 days (Caco_2_: 1.17±0.04 and 1.2 ± 0.04; SW48: 1.1 ± 0.02 and 1.16 ± 0.02, respectively) (Figure 3). 

***Western blot analysis of signaling proteins***

**Figure 4:**
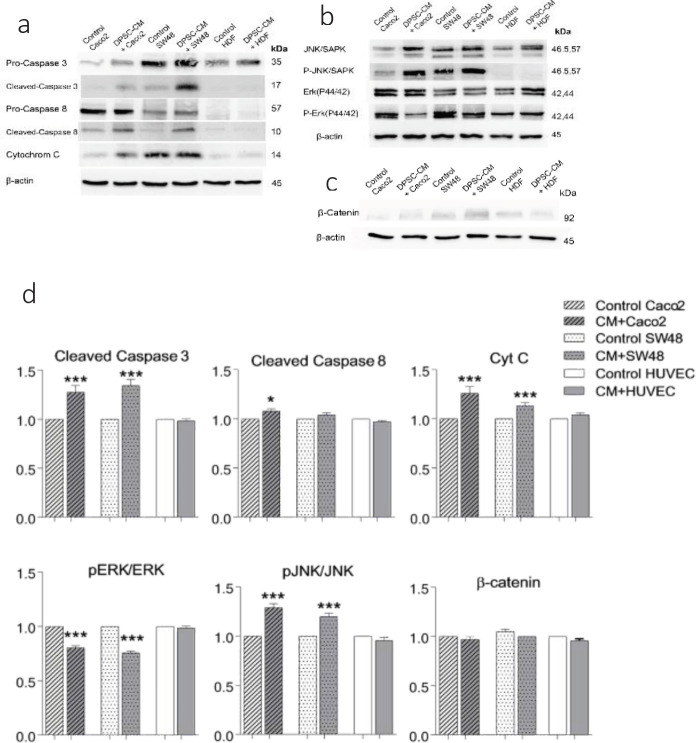
Effect of CM-40K-4d on the level of apoptotic signaling proteins: A. caspase 3, caspase 8, cyt C. B. MAPKinase signaling proteins, and C. β-catenin protein by western blot analysis. Cells (3×106) were treated with or without CM-40K-4d for 3 days. D. Expression level of apoptosis proteins was analyzed by using Gel-pro Analyzer V.6.0 Gel Analysis Software. The expression levels were normalized by the density of β-actin protein level. The data are expressed as mean ± SD of three independent experiments, *P<0.05 and ***P<0.001 compared with the control

CM: Conditioned medium; MAPKinase: Mitogen-activated protein kinase

To determine the mechanism of inhibitory effects of DPSC-CM on CRC cells, the expression of apoptotic proteins (pro and cleaved-caspase 3, pro and cleaved-caspase 8, cytochrome C, ERK (P44/42), p-ERK (P44/42), JNK/SAPK, p-JNK/SAPK, and β-Catenin) were compared in the Caco_2_, SW48 and HUVEC cells treated with DPSC-CM or FBS free medium. The results revealed that treatment with CM-40K-40d for 3 days significantly increased the levels of cleaved caspase 3 and cytochrome C in both CRC cells and cleaved-caspase 8 only in Caco_2_. Furthermore, DPSC-CM decreased p-ERK (P44/42) to ERK (P44/42) ratio and significantly increased p- JNK/SAPK to JNK/SAPK ratio of MAPKinase pathways in CRC cells compared with the untreated control group. Variation in the expression level of β-catenin between different samples was not significant (Figure 4). 

***Effect of indirect co-culture of DPSCs and CRC cells on the apoptosis of CRC cells***

**Figure 5:**
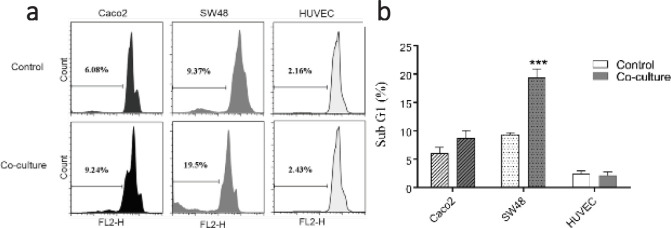
Indirect effect of DPSC on the apoptosis of CRC cells. A. PI staining and flow cytometry analysis of indirect co-culture of DPSCs with cancer and normal cells. B. Data are expressed as mean ± SD in triplicate, ***P<0.001 compared with the control

DPSCs: Dental Pulp stem cells; CRC: Colorectal cancer

To study the indirect effect of DPSCs on the apoptosis of cancer cells, DPSCs were seeded in the upper chamber and Caco_2_, SW48, and HUVEC cells were seeded in the lower chamber of a 24-Transwell plate (0.4 µm pore size) at a ratio of 1:1. After 3 days, the cells in the lower chamber were removed and analyzed by PI apoptosis assay (Figure 5). The result of the indirect co-culture assay indicated that DPSC significantly increased the apoptosis of SW48 cells (20.45%± 1.48) in comparison with control (10.33%± 1.36).

***The effects of CRC cells in the DPSCs homing***

**Figure 6:**
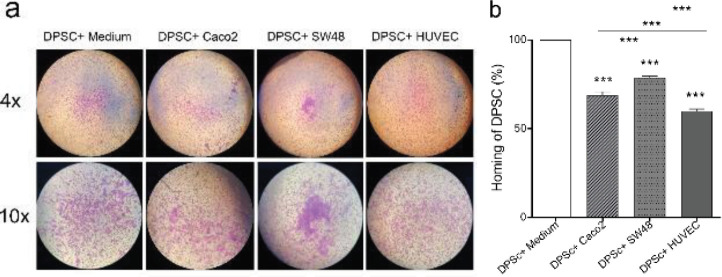
Evaluation of the homing potential of DPSCs to CRC cells* in vitro*. DPSCs were cultured in the upper chamber and co-cultured with Caco_2_, SW48, and HDF in the lower chamber of a 24-Transwell plate. A. migrated DPSCs to cancer and normal cells in a Transwell plate (pore size: 8 µm). Magnification 4× and 10×. B. percent of migrated DPSCs was normalized to the crystal violet absorbance unit of control at 570 nm and compared with the control. Data are expressed as mean ± SD in triplicate. ***P<0.001

DPSCs: Dental Pulp stem cells; CRC: Colorectal cancer

To investigate the homing potential of DPSCs to CRC cells* in vitro,* DPSCs were cultured in the upper chamber and co-cultured with Caco_2_, SW48, and HUVEC in the lower chamber of the Transwell plate. After 3 days, the migration of DPSCs to the underside of the inserts was analyzed after staining with crystal violet (Figure 6). Based on the results, DPSCs demonstrated significant homing (migration) to Caco_2_ and SW48 (68.84%±1.97 and 78.72%±1.14, respectively) compared with HUVEC (59.97%±1.1). Furthermore, DPSCs migrated to SW48 (78.72%±1.14) significantly more than Caco_2_ (68.84%±1.97), and DPSCs migrated to Caco_2_ (68.84%±1.97) more than HUVEC (59.97%±1.1).

***The effect of DPSCs on the invasion ability of CRC cells***

**Figure 7:**
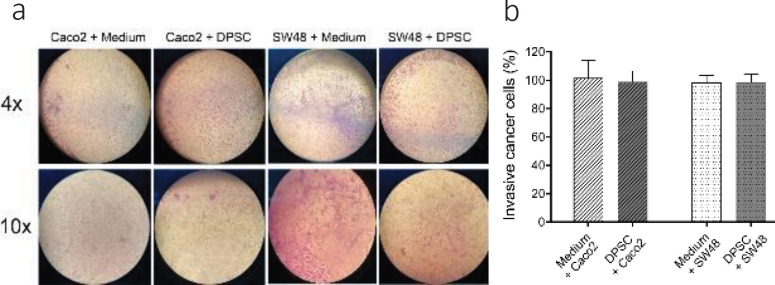
Effects of indirect co-culture of DPSCs and CRC cells on the invasive ability of CRC cells. A. Image of migrated cells stained with crystal violet underside of the inserts. Magnification, 4× and 10×. B. Percent of metastatic cancer cells was normalized to the crystal violet absorbance unit at 570 nm and compared with the control. Data represented no significant differences in invasion of cancer cells between the control group and the co-culture group and are expressed as mean ± SD in triplicate

DPSC-CM: Dental pulp stem cell-conditioned medium; CRC: Colorectal cancer

To detect the inhibitory effect of DPSCs on Caco_2_ and SW48 cell invasion *in vitro*, cancer cells were seeded in the upper chamber of Transwell Plates and co-cultured with DPSCs or FBS free medium in the lower chamber. After 3 days, the cancer cells that moved to the underside of the inserts were analyzed by staining with crystal violet (Figure 7). The results showed that DPSDs did not have any effect on the invasion ability of CRC cells. 

**Table 1:** Schedule and cell density for DPSC-CM collection

	Density (cell count)			
Days of incubation	5000 (5 K)	10000 (10 K)	20000 (20 K)	40000 (40 K)
2	CM-5k-2d	CM-10k-2d	CM-20k-2d	CM-40k-2d
3	CM-5k-3d	CM-10k-3d	CM-20k-3d	CM-40k-3d
4	CM-5k-4d	CM-10k-4d	CM-20k-4d	CM-40k-4d

DPSC-CM: Dental pulp stem cell-conditioned medium; CM: Conditioned medium

**Figure 8:**
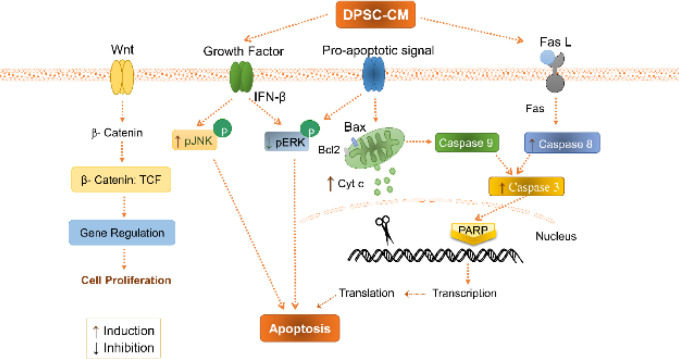
Schematic representation of the suppressive role of DPSC-CM on CRC cells. DPSC-CM induced apoptosis through an increase of pJNK/JNK and a decrease of pERK/ERK pathways. DPSC-CM increased cytochrome C in the intrinsic pathway, cleaved caspase 8 in the extrinsic pathway and cleaved caspase 3 in the apoptosis signaling pathway. It had no effect on β-catenin in the WNT pathway in CRC cells

DPSC-CM: Dental pulp stem cell-conditioned medium

## Discussion

According to our findings, the viability of CRCs was decreased when exposed to DPSC-CM and the rate of apoptosis of CRC cells was increased significantly. Cytochrome C, phospho-JNK/SAPK to JNK/SAPK ratio, cleaved-caspase 8 and 3 were all increased in treated CRC cells with DPSC-CM, and phospho-ERK (P44/42 MAPK) to ERK (P44/42 MAPK) ratio was decreased while minimal change was seen in normal cells. Additionally, the homing of DPSCs towards Caco_2_ and SW48 cells was verified.

Dental pulp MSCs, isolated from pulp tissue, have an embryonic origin and maintain their stemness properties during passages (37). It has been shown that the conditioned medium of MSCs (MSC-CM) could inhibit immune responses and tumor progression. For example, it has been reported that the proliferation of melanoma and human breast cancer cells could be inhibited by adipocyte-MSC-CM without cell-to-cell contact (10, 30). Also, it has been shown that high-density (40K) adipocyte-MSC increased the expression of interferon IFN-β, TRAIL, and tumor necrosis factor in H460 human lung cancer cells, significantly, induced apoptosis and suppressed the growth of human lung tumors (16). As there are no previous reports on the effects of DPSC and DPSC-CM on cancer cells, in the present study, the cytotoxic and apoptotic effects of DPSCs and DPSCs-CM on CRC cells have been evaluated. Interestingly, treatment with DPSC-CM collected from 40000 DPSCs/cm^2^ cultured for 4 days (CM-40K-4d) could effectively inhibit the growth of CRC cells and induce apoptosis. CM-40K-4d induced apoptosis significantly in both Caco_2_ and SW48 compared with the control. Moreover, DPSC-CM decreased the viability of cancer cells beginning at 20K in SW48 and 40K in Caco_2_ after 3 days. The chromosomal instability gene expression of Caco_2,_ which causes disruption of tumor suppressor genes may be the reason for less susceptibility of Caco_2_ to DPS-CM in comparison with SW48 cells (21). No significant toxic and apoptotic effect of DPSC-CM was seen on normal cells. However, 5-FU had significantly induced apoptosis on non-cancer cells. 

As Caco_2_ and SW48 cells are not very invasive, no significant changes were seen in the wound area of untreated cells after 3 days. Besides, cells treated with both CM-40k-3d and CM-40k-4d could significantly increase the area of scratch after 3 days in both CRC cells. Enlargement of the wound surface showed DPSC-CM could inhibit cell migration also cause an increase in the wound area due to inhibition of cell growth and induction of cell death. This finding supports the result of the viability assay in the present study. 

Western blotting results showed that treatment of CRCs with CM-40K-4d for 3 days elevated the expression level of cleaved caspase 3 and cytochrome C proteins in both Caco_2_ and SW48, and cleaved-caspase 8 in Caco_2_ in the apoptosis pathway. So, it seems that both intrinsic and extrinsic pathways of apoptosis are involved in apoptosis induction (Figure 8) (38-40).

In addition, DPSC-CM decreased the phosphorylation of ERK44/42 and increased the phosphorylation of JNK/SAPK46/54 in CRC cells, which is a sign of apoptosis induction in cancer cells (38). The ERK MAPKinase molecule involves in the progression, pathogenesis, and oncogenesis of CRC in response to extracellular stimulants. Deactivation of ERK pathway enhances cancer cell death and could be a target to inhibit the development of the CRC (38, 41, 42). Activation of the JNK pathway is associated with transformation in the growth factor-mediated pathways and many oncogenes are involved in both apoptosis and survival signaling. Some evidence suggested that only the ERK pathway signaling, but not the JNK pathway, is a major regulator of cell migration, proliferation and differentiation in CRC. Accordingly, the induction of JNK and inhibition of ERK activity leads to apoptosis and differentiation of intestinal cells, which could be considered as a new strategy to prevent CRC development and a potential target for cancer therapy (7, 43, 44). Here we showed DPSC-CM prevents the development of CRC through modulation of MAPKinase pathway by inhibition of ERK phosphorylation and phosphorylation of JNK (Figure 8). 

The expression of β-catenin, the main protein in WNT pathway, was not significantly changed in Caco_2_ and SW48 under treatment with DPSC-CM. The Wnt/β-catenin pathway is one of the crucial signaling cascades in cellular proliferation and differentiation in many cancers such as CRC (45, 46). Although MSCs suppress the growth of CRC cell lines via inhibition of the Wnt/β-catenin pathway, it seems the variety of CRC cell lines shows different responses depending on the stage of the cancer progress (Figure 8) (11).

Significant apoptosis induction in SW48 cells in the indirect co-culture method confirms at least a partial role of apoptosis in DPSC cytotoxicity for cancer cells. Likewise, DPSCs significantly migrated to SW48 more than Caco_2_ and it can be a reason that DPSCs are more effective on SW48 than Caco_2_ cells. Based on the literature, cancer cells release regulatory molecules that stimulate the surrounding stromal cells to proliferate and migrate into the tumor (47). Tumor-derived biomarkers have the potential to attract MSCs. Targeted homing of the MSCs into the primary and metastatic tumor has been represented in most types of cancer. It has been reported that targeted homing of MSCs toward the microenvironment of tumors including primary and metastatic tumors prevents the activity of tumor cells and tumor development by inhibition of Wnt and AKT signaling (48). Accordingly, MSCs had the potential to deliver the vehicles for cancer therapy because of homing capacity and migration toward the tumor sites in glioma, renal cell carcinoma, and lung and prostate tumors (49, 50). 

## Conclusion

The findings of the present study show that 4 days of exposure to high-density DPSC-CM could induce apoptosis in CRC cells with negligible effect on the control HUVEC. The possible mechanisms appear to be through a reduction in phosphorylation of ERK, induction of JNK phosphorylation, and activation of both intrinsic and extrinsic pathways of apoptosis. Homing ability of DPSCs toward Caco_2_ and SW48 cells was also confirmed. Therefore, DPSC-CM could effectively inhibit the growth of CRC cells and may be considered as a novel and non-invasive strategy to fight against tumor development with acceptable homing potential. 

## Funding Information

This work was supported by the Research Council of Mashhad University of Medical Sciences, Iran (grant no. 951441). This project was done as a part of a PhD thesis by Elham Nikkhah.

## Authors’ Contributions

EN, FK, MAR, and ZTN Study conception and design; EN Data analysis and draft manuscript preparation; FK and ZTN Critical revision of the paper; ZTN Supervision of the research; EN, FK, MAR, and ZTN Final approval of the version to be published.

## Conflicts of interest

The authors declare that there are no conflicts of interest.
